# Effects of Infrared Treatment on Some Constituents and Functional Properties of Chia Seed

**DOI:** 10.1002/fsn3.70308

**Published:** 2025-06-03

**Authors:** Meltem Laçin, Arzu Başman

**Affiliations:** ^1^ Food Engineering Department Hacettepe University Ankara Turkey

**Keywords:** antioxidant capacity, chia, functional properties, infrared treatment, phenolic profile, total flavonoid content

## Abstract

Infrared treatment has become increasingly popular in the food industry due to its advantages over conventional heating. The aim of this study is to enhance health beneficial components and functional properties of chia seeds by using infrared treatment at different powers (700, 900, 1100 W) and times (25 and 50 min). Infrared treatment caused a significant, gradual increase in the total phenolic content and antioxidant capacity of the chia samples. Total flavonoid and total dietary fiber content increased at 700 and 900 W treatments. Chlorogenic acid, ferulic acid (except 1100 W–50 min treatment), rutin, and quercetin (only at 700 W–50 min & 900 W–25 min treatments) increased, whereas caffeic and p‐coumaric acid decreased by infrared treatment. FTIR spectra for infrared treated samples have all specific peaks reported in the literature for chia. Slight changes were observed in peak intensities of infrared treated samples. Infrared treatment enhanced emulsion activity (up to 900 W–25 min) and stability (up to 700 W–50 min) but adversely affected the water and oil holding capacities. Infrared treatment of chia seeds enhanced the extractability of health beneficial constituents (e.g., phenolics and flavonoids) probably due to thermal breakdown of cellular components, and this may result in a health promoting effect. Utilization of infrared treated chia seeds as a raw material in value‐added food products is promising.

## Graphical abstract

Infrared treatment enhanced the antioxidant capacity, total flavonoid, and total phenolic content of the chia and enabled the extractability of rutin and ferulic acid. Infrared treatment adversely affected water and oil holding capacities while improving emulsion activity (at 700 W and 900 W–25 min) and stability (at 700 W). Infrared treatment which improves the amount of health beneficial constituents of chia is promising for utilization of chia as raw material in value‐added food products.

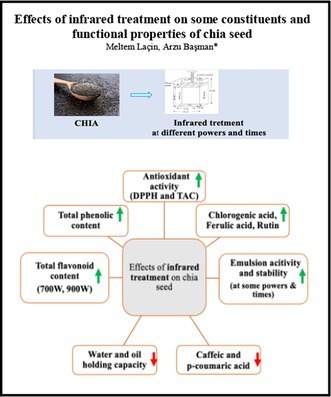

## 1 Introduction

In recent years, there has been a growing trend in the consumption of chia seeds. Chia ( *Salvia hispanica* L.) is a pseudocereal with small, oval‐shaped seeds. Chia seeds contain 15%–25% protein, 30%–33% fat, 18%–30% total dietary fiber, 26%–41% carbohydrates, minerals, and vitamins (Hrnčič et al. 2020; Julio et al. 2019). About 60% of the oil fraction of chia seeds is omega‐3 while 20% of the oil fraction is omega‐6 (Fernandes et al. 2020). Chia seeds contain phenolic compounds, such as chlorogenic acid, caffeic acid, rutin, p‐coumaric acid, rosmarinic acid, ferulic acid, quercetin, kaempferol, catechin, etc. Chia seeds are a potential source of antioxidants because of their phenolic compounds (Hrnčič et al. 2020; Ghafoor et al. 2022). The rich composition of chia makes it a unique seed and has an effect on reducing the risk for coronary heart disease, diabetes mellitus type 2, and several types of cancer. Chia seeds, being gluten‐free, are also a suitable option for individuals with celiac disease (Ullah et al. 2016). Chia flour can be used in flour blends to enhance the nutritional value and sensory qualities of cereal products, such as chips, wheat bread, and biscuits (Coorey et al. 2012; Divyashree et al. 2016; Romankiewicz et al. 2017).

Infrared treatment has become increasingly popular in the food industry because of its advantages over conventional heating, such as improved energy efficiency, high heat transfer coefficient, short processing time, and uniform temperature distribution (Nowak and Lewicki 2004; Rojas and Augusto 2018; Sakare et al. 2020). Infrared treatment has been applied in various processes, such as roasting, drying (grains, fruits, vegetables, etc.), extraction (polyphenols, flavonoids, etc.), cooking, baking, enzyme inactivation, pasteurization, and sterilization (Rastogi 2012; Yalcin and Basman 2015; Sakare et al. 2020; Manyatsi et al. 2023). Infrared was used in the production of noodles (Basman and Yalcin 2011), heat‐moisture treated starch (Ismailoglu and Basman 2015, 2016), bulgur (Savas and Basman 2016), bread (Panirani et al. 2023), and cake (Sumnu et al. 2005). Earlier research has shown that infrared treatment might assist in releasing some nutritional components by breaking down cell walls and cellular constituents. Infrared treatment increased the total phenolic content of soybean (Yalcin and Basman 2016), peanut (Bagheri et al. 2016), rice bran (Irakli et al. 2018) and green pea (Kaveh and Abbaspour‐Gilandeh 2020). Infrared treatment also increased the antioxidant activity of rice bran (Irakli et al. 2018), green pea (Kaveh and Abbaspour‐Gilandeh 2020) and hazelnut (Binello et al. 2018). Studies have shown that infrared treatment has advantages during the cooking and roasting of foods, particularly enhancing the extraction of phenolic and flavonoid compounds.

Roasting, as a taste‐ and aroma‐enhancing thermal process, has been applied to chia seeds to improve their nutritional quality and promote their use in value‐added food products, thereby increasing the consumability of chia seeds. Since roasting temperatures and times vary across different studies, both increases and decreases in the nutritional constituents of the chia have been reported. Some studies have reported that roasting caused increases in total phenolic content (Ghafoor et al. 2018; Hatamian et al. 2020; Mohamed Ahmed et al. 2024), total flavonoid content (Ghafoor et al. 2018), and antioxidant activity (Hatamian et al. 2020) of chia seeds. However, decreases were also reported for caffeic acid, quercetin, rutin, ferulic acid, p‐coumaric acid, total flavonoid content (Mohamed Ahmed et al. 2024), and antioxidant activity (Ghafoor et al. 2020) of roasted chia seeds.

In literature, infrared was used only in a research about chia investigating the effects of infrared treatment on chia seed oil composition (Sundar et al. 2023). To the best of our knowledge, in literature there is no study about the effects of infrared treatment on other constituents of chia seed. Therefore, in this study, chia seeds were infrared treated at different powers (700, 900, 1100 W) and times (25 and 50 min). The effects of infrared treatment on protein, ash, color, total dietary fiber, total phenolic content, total flavonoid content, phenolic profile, antioxidant capacity, FTIR, and functional properties of the chia seeds were investigated.

## 2 Materials and Methods

### 2.1 Material

Chia seeds ( *Salvia hispanica* L.) were purchased from “Yayla Agro Sanayi ve Ticaret A.S.” (Ankara, Turkey). Quercetin (Sigma PHR1488, USA), caffeic acid (Sigma C0625, USA), chlorogenic acid (Cayman Chemicals‐70930, USA), Trolox (Sigma 238813, USA), rutin hydrate (Sigma R5143, USA), ferulic acid (Sigma PHR1791, USA), p‐coumaric acid (Sigma C9008, USA) were used as standards in the study.

### 2.2 Infrared Treatment

In the present study, a laboratory‐scale infrared equipment, fitted with 12 halogen lamps (150 W each) (Infrared, BR125 IR; Philips, Eindhoven, The Netherlands) and two aeration channels in a closed drying chamber with aluminum reflectors on the walls, was used. The wavelength spectrum of halogen lamps was 0.2–4 μm, with a pronounced peak at approximately 1 μm. The distance between the tray and light source was set to 20 cm.

Since in preliminary studies, infrared treatment at 1100 and 1200 W for 60 min resulted in burnt chia seeds, in the study, infrared treatment was applied at 700, 900, and 1100 W for 25 and 50 min. Infrared treatment at each power and time was carried out as seven replicates. The moisture content of the control chia sample was 6.89%. For each replication, 140 g chia samples were spread on a wooden tray with a homogeneous thickness of 2–3 mm. During the infrared treatment at each power and time, the surface temperatures of the samples were measured every 5 min at four different points by an IR thermometer (Raytek MX6, Germany). The average surface temperatures of the chia samples infrared treated at different powers and times were 113.5°C (700 W–25 min), 119.4°C (700 W–50 min), 139.6°C (900 W–25 min), 148.3°C (900 W–50 min), 160.8°C (1100 W–25 min) and 168.6°C (1100 W–50 min). The ambient temperature inside the infrared equipment was measured with a thermocouple. Seven replicates for each infrared power–time treatment were combined in a single large batch. The samples were ground using a Multipurpose Disintegrator (IC‐04A, China) and sifted through an 850 μm sieve. The ground chia sample (< 850 μm) was stored at +4°C until analysis.

### 2.3 Chemical Composition

Moisture, ash, and protein contents of control and infrared treated chia samples (< 850 μm) were determined by using methods of American Association of Cereal Chemists (AACC) Method No 44‐19, Method No 08‐01, and Method No 46‐12, respectively (AACC 2010).

The total dietary fiber content of defatted chia samples was determined by using the Megazyme Total Dietary Fiber Assay Kit (Megazyme International Ireland Ltd., Ireland). Since the fat content of chia is greater than 10%, fat was removed. The ground chia (< 850 μm) was mixed with hexane at a ratio of 1:5 (w/v) and stirred with a magnetic stirrer for 1 h, and hexane was removed at room temperature. The aim of the procedure is to remove starch and protein by using α‐amylase, protease, and amyloglucosidase by incubation at different conditions. The results were given on a dry basis as the average of two values.

### 2.4 Color

Color measurement (L*, a*, b*) was carried on ground chia samples using the CIE L*a*b* color system (Minolta Spectrophotometer CM‐3600d, Japan), where L* is lightness–darkness, a* is redness–greenness, and b* is yellowness–blueness. The results were given as the average of three values. The color difference (Δ*E*) is calculated by the following equation:

$$∆E=\left(\sqrt{{\left(\Delta L^{*}\right)}^{2}+{\left(\Delta a^{*}\right)}^{2}+{\left(\Delta b^{*}\right)}^{2}}\right)$$

Δ*E* was calculated in order to determine the effects of the infrared treatment at different powers and times on the color value of the chia samples. Δ*E* is a single value that takes into account the differences between the L*, a*, or b* of the infrared treated and the control chia sample.

### 2.5 Fourier Transform Infrared Spectroscopy (FTIR)

Control and infrared treated chia samples were characterized by using Attenuated Total Reflectance‐Fourier Transform Infrared Spectroscopy (ATR‐FTIR) (Thermo Scientific, Nicolet iS50, Waltham, MA). Analysis was carried out as 16 scanning frequencies over a wavenumber range of 4000–400 cm^−1^ with 4 cm^−1^ resolution.

### 2.6 Extraction

Extraction was carried out by using Beltrán‐Orozco et al. (2020) method with some modifications. Ethanol (80%, 10 mL) was added to 1 g (db) chia and mixed for 4 h at room temperature. The samples were centrifuged at 6000 *g* at 4°C for 15 min. The extract was filtered through Whatman No. 1. The extract was used in the determination of total phenolic content, total flavonoid content, and % radical scavenging activity (DPPH).

### 2.7 Total Phenolic Content

Total phenolic content was determined by using the Folin–Ciocalteu method (Singleton et al. 1999) with some modifications described by Beltrán‐Orozco et al. (2020). Fresh chia extract (0.06 mL) was mixed with 1.54 mL distilled water, and 100 μL Folin‐Ciocalteu reagent was added, vortexed for 10 s, and left at room temperature for 3 min. Then, Na_2_CO_3_ solution (300 μL, 10% w/v) was added, vortexed for 10 s, and rested at room temperature for 60 min in a dark place. The absorbance was measured at 765 nm. The calibration curve was plotted using standard gallic acid (0–300 ppm). The results were given as the average of four values and expressed as mg GAE/g dry sample.

### 2.8 Total Flavonoid Content

Total flavonoid content was determined by using the method described by Scapin et al. (2016). Two hundred and fifty microliter chia extract was mixed with 1250 μL distilled water. Then, 75 μL sodium nitrite (NaNO_2_) solution (5%) was added, and the mixture was allowed to stand for 5 min. Then, 150 μL aluminum chloride solution (10%) was added, and the mixture was left to stand for 5 min. Five hundred microliter sodium hydroxide (1 M) and 775 μL distilled water were added and mixed thoroughly. The absorbance was measured immediately at 510 nm. The calibration curve was plotted using standard quercetin (0–160 ppm). The results were given as the average of three values and expressed as mg quercetin/g dry sample.

### 2.9 Antioxidant Capacity

#### 2.9.1 Radical Scavenging Activity (DPPH Assay)

Radical scavenging activity (%) was determined by using the method described by Laczkowski et al. (2018). DPPH (2.2‐Diphenyl‐1‐picrylhydrazyl) solution was prepared with 80% ethanol at a concentration of 6 × 10^−5^ M. DPPH solution (3.9 mL, 6 × 10^−5^ M) was added to 0.1 mL chia extract, vortexed for 10 s, and kept in a dark place for 30 min.

For control sample, 3.9 mL DPPH solution was added to 0.1 mL ethanol (80%). The absorbance was measured at 515 nm against 80% ethanol (blank). The radical scavenging activity (%) was calculated according to the equation given below, and the results were given as the average of three values.

$$\text{Radical scavenging activity}\;\left(\%\right)=\left(1-\frac{\text{absorbance of the sample}}{\text{absorbance of the control}}\right)\times 100$$

#### 2.9.2 Total Antioxidant Capacity (QUENCHER‐CUPRAC Method)

QUENCHER‐CUPRAC method described by Tufan (2012) was used in order to determine the total antioxidant capacity of chia samples. Chia (100 mg, db) was first mixed with 900 mg (db) cellulose in order to obtain a ratio of 1/10 (w/w). Chia (10 mg) was taken from this mixture and mixed with 4.1 mL CUPRAC reagent solution. CUPRAC reagent solution was prepared by adding copper (II) solution (1 mL), neocuproin solution (1 mL), ammonium acetate solution (1 mL) and water: ethanol (1.1 mL, 1:1 v/v). Following 1 min vortexing and 30 min shaking, the sample was centrifuged at 6654 *g* for 2 min and filtered through a 0.45 μm micro‐filter (Chromafil GF/PET‐45/25, Macherey Nagel, Germany). Absorbance was measured at 450 nm. Total antioxidant capacity was calculated from a standard curve of Trolox (1.22 × 10^−5^ – 7.32 × 10^−5^ M).

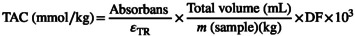


*A*: sample absorbance measured at 450 nm; *ε*_TR_ (L/mol cm): molar absorption coefficient of Trolox in CUPRAC method (Coefficient of *x* in the equation of the calibration curve for Trolox standard); Total volume: total volume of CUPRAC solution (4.1 mL); DF: Dilution factor; m: amount of sample taken.

### 2.10 Phenolic Profile

Extraction: Chia (1 g, db) was mixed with 80% methanol for 6 h. The sample was centrifuged at 6000 *g* for 15 min at 4°C. The extract was filtered through Whatman No. 1 and then a 0.45 μm micro‐filter (Chromafil GF/PET‐45/25, Macherey Nagel, Germany) and analyzed immediately (Abdel‐Aty et al. 2021).

Caffeic acid, chlorogenic acid, rutin, p‐coumaric acid, ferulic acid, and quercetin content of chia samples was determined by using the modified method of Pająk et al. (2019). The chromatographic analyses were carried out in HPLC (Agilent Technologies, Waldbronn, Germany) equipped with a Multiple Wavelength Detector (MWD) (Agilent Technologies, Waldbronn, Germany).

The sample was analyzed on a C18 column (250 mm × 4.6 mm × 5 μm; Supelco, Germany) at a temperature of 30°C. Chromatographic separation was performed at a flow rate of 1.0 mL/min with gradient elution by using two solvents for the mobile phase: solvent A‐2% acetic acid and solvent B‐acetonitrile. The gradient system started from 97% B at 0 min, to 92% B at 10 min, 85% B at 20 min, 80% B at 30 min, 70% B at 40 min, and 60% B at 50 min. The injection volume was 10 μL. Chlorogenic acid was detected at *λ* = 280 nm and caffeic acid, p‐coumaric acid, rutin, ferulic acid, and quercetin were detected at *λ* = 320 nm. The results were given as the average of four values and expressed as mg/100 g dry sample. Standards for caffeic acid (6.25–10 μg/mL), chlorogenic acid (2–20 μg/mL), rutin (2–42 μg/mL), p‐coumaric acid (0.2–4.2 μg/mL), ferulic acid (0.25–5.5 μg/mL), and quercetin (5–30 μg/mL) were prepared by using methanol (80%).

### 2.11 Functional Properties

#### 2.11.1 Water Holding Capacity

Water holding capacity of control and infrared treated chia samples was determined by using the method of Alfredo et al. (2009). Ten microliter distilled water was added to chia (1 g, db) during continuous stirring for 1 min at room temperature and then centrifuged at 2200 *g* for 30 min. The water holding capacity was expressed as the grams of retained water/gram of dry sample. The results were given as the average of three values.

#### 2.11.2 Oil Holding Capacity

Oil holding capacity of control and infrared treated chia samples was determined by using the method of Alfredo et al. (2009). Chia (1 g, db) was mixed with 10 mL corn oil for 1 min at room temperature and then centrifuged at 2200 *g* for 30 min. The oil holding capacity was expressed as the grams of retained oil/gram of dry sample. The results were given as the average of three values.

#### 2.11.3 Emulsion Activity and Stability

Emulsion activity of control and infrared treated chia samples was determined by using the method of García‐Salcedo et al. (2018) with some modifications. Chia (1 g, db) was mixed with 20 mL distilled water and homogenized (IKA Ultraturrex, Germany) at 10,000 rpm for 25 s. Then, 7 mL corn oil was added and homogenized for 25 s. After centrifugation at 1664 *g* for 9 min, the height of the emulsion layer was measured. The emulsion activity (%) was calculated by using the equation given below. The results were given as the average of three values.

$$\text{Emulsion activity}\;\left(\%\right)=\frac{\text{Height of the emulsified layer}\;}{\;\text{Height of the tube content}}\times 100$$

Emulsion stability of control and infrared treated chia samples was determined by using the method of Betancur‐Ancona et al. (2004) with some modifications. The tubes for which the emulsion activity was calculated were heated at 80°C for 30 min, then cooled to room temperature and centrifuged at 1664 *g* for 10 min. The height of the emulsion layer was measured. The emulsion stability (%) was calculated by the equation given below. The results were given as the average of three values.

$$\text{Emulsion stability}\;\left(\%\right)=\frac{\text{Height of the emulsified layer}\;}{\;\text{Height of the tube content}}\times 100$$

### 2.12 Statistical Analysis

The results were evaluated by using one‐way analysis of variance (ANOVA) (SPSS 23.0, USA). When significant differences were found, the Duncan comparison test was used to determine the differences between means. A *p* value of less than 0.05 was considered statistically significant. The standard deviation values for some results were calculated by using MS Excel (MS Excel; Microsoft Corp., USA).

## 3 Results and Discussion

### 3.1 Chemical Composition

Chemical composition of the infrared treated chia samples is given in Table 1. The protein content of control chia was found to be 22.42% (db), which is similar to the values (17%–24%) reported in the literature for chia seeds (Ayerza and Coates 2005; Coelho and Salas‐Mellado 2014; Segura‐Campos et al. 2014). Chia has a higher protein content as compared to traditional grains, such as wheat, corn, rice, oats, and barley (Julio et al. 2019). Infrared treatment did not cause a significant change in the protein content of the chia samples (Table 1). The ash content of the control chia was 4.76% (db). Infrared treatment caused slight but significant changes in the ash content of the chia samples.

**TABLE 1:** Chemical composition of the infrared treated chia samples.

Sample	Moisture (%)	Protein (%, db)	Ash (%, db)	Total dietary fiber (%, db)
Control	6.89 ± 0.06^a^	22.42 ± 0.10^a^	4.76 ± 0.01^c^	36.61 ± 0.12^e^
700 W–25 min	3.23 ± 0.01^b^	21.77 ± 0.56^a^	4.77 ± 0.01^b^	37.07 ± 0.12^d^
700 W–50 min	1.53 ± 0.03^c^	21.76 ± 0.15^a^	4.73 ± 0.00^d^	38.94 ± 0.18^c^
900 W–25 min	1.17 ± 0.00^d^	21.62 ± 0.25^a^	4.72 ± 0.00^d^	40.83 ± 0.29^a^
900 W–50 min	1.05 ± 0.04^e^	21.33 ± 0.01^a^	4.80 ± 0.01^a^	39.95 ± 0.06^b^
1100 W–25 min	0.51 ± 0.02^f^	21.73 ± 0.85^a^	4.75 ± 0.01^c^	32.39 ± 0.03^f^
1100 W–50 min	0.49 ± 0.01^f^	21.36 ± 0.64^a^	4.76 ± 0.01^bc^	32.68 ± 0.06^f^

*Note:* Values followed by the same letter in the same column are not significantly different (*α* = 0.05). Values are the means of two replicates.

Abbreviation: db, dry basis.

The total dietary fiber content of the control chia sample was 36.61% (db). In literature, the total dietary fiber content of chia was reported as 374.4 g/kg seed (Scapin et al. 2016) and 39.94–41.41 g/100 g seed (db) for cv. Jalisco and 36.97–38.79 g/100 g seed (db) for cv. Sinaloa (Reyes‐Caudillo et al. 2008). Higher total dietary fiber content was reported for chia compared to that of wheat, barley, rye, quinoa, and corn (Prasadi and Joye 2020). The total dietary fiber contents of the infrared treated chia samples (except chia samples infrared treated at 1100 W) were significantly higher than that of the control sample. Among all samples, the highest total dietary fiber content (40.83%, db) was obtained for the chia sample infrared treated at 900 W–25 min. Similar fluctuations in total dietary fiber content after infrared treatment were also reported by Liu et al. (2022) for infrared treated lentil samples.

### 3.2 Color Values

L*, a*, and b* color values of the chia samples were between 42.65–44.50, 1.26–1.75, and 2.15–2.98, respectively (Table 2). Infrared treatment did not cause substantial changes in the color values of the chia samples. A minor decrease in L* (lightness) color value was observed as the infrared power increased. As compared to control, the decrease in L* value was significant for the infrared treated chia samples (except chia sample infrared treated at 700 W–25 min). Infrared treatment caused slight but significant changes in a* and b* color values.

**TABLE 2:** Color values of the infrared treated chia samples.

Sample	L*	a*	b*	Δ*E*
Control	44.50 ± 0.63^a^	1.52 ± 0.03^c^	2.84 ± 0.04^b^	—
700 W–25 min	44.30 ± 0.29^a^	1.30 ± 0.06^d^	2.67 ± 0.03^c^	0.80 ± 0.11^a^
700 W–50 min	43.46 ± 0.05^b^	1.26 ± 0.04^d^	2.50 ± 0.02^d^	1.16 ± 0.55^a^
900 W–25 min	43.47 ± 0.28^b^	1.47 ± 0.02^c^	2.25 ± 0.05^e^	1.20 ± 0.46^a^
900 W–50 min	43.33 ± 0.50^bc^	1.75 ± 0.05^a^	2.24 ± 0.06^e^	1.43 ± 0.72^a^
1100 W–25 min	43.04 ± 0.62^bc^	1.27 ± 0.06^d^	2.15 ± 0.06^f^	1.79 ± 0.87^a^
1100 W–50 min	42.65 ± 0.27^c^	1.60 ± 0.01^b^	2.98 ± 0.02^a^	1.86 ± 0.37^a^

*Note:* Values followed by the same letter in the same column are not significantly different (*α* = 0.05). Values are the means of three replicates.

Δ*E* values increased as the infrared power and time increased. Among all samples, Δ*E* for the 1100 W–50 min chia sample was found to be the highest (1.86). Similar to our results, a decrease in the L* color value was also reported for roasted chia samples (Mohamed Ahmed et al. 2024) and infrared treated soybean samples (Yalcin 2011). The increase in Δ*E* value may be due to the dehydration of carbohydrates and the Maillard reaction, both of which vary depending on the temperature and time applied during the process (Mohamed Ahmed et al. 2024).

### 3.3 Fourier Transform Infrared Spectroscopy (FTIR)

**FIGURE 1:**
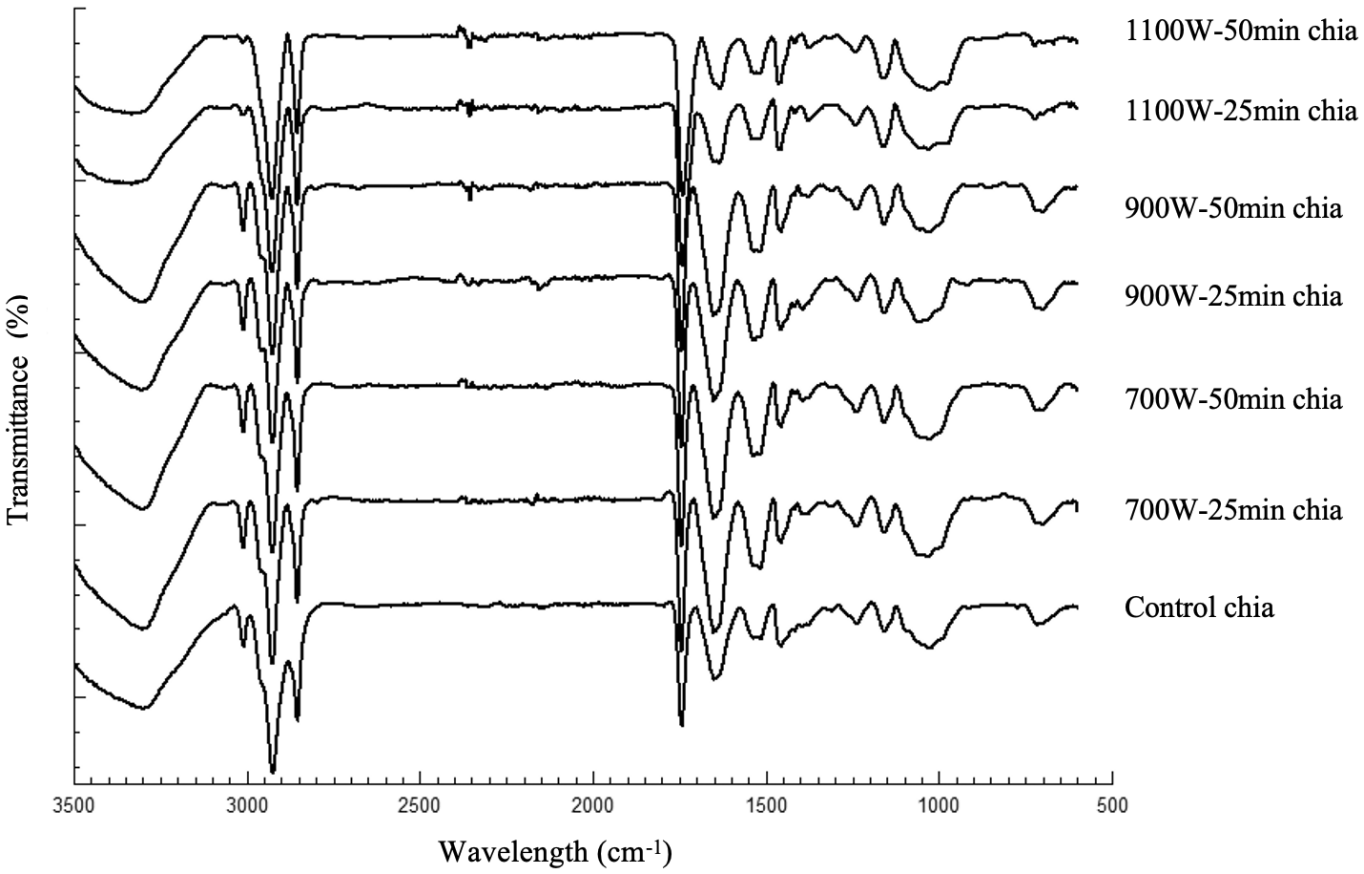
FTIR spectra of control and infrared treated chia samples.

FTIR spectrums (Figure 1) for control and infrared treated chia samples include all the specific peaks reported in literature for chia, but slight changes were observed in the intensity of some of the peaks.

In the present study, characteristic absorption peaks of the chia samples observed around 3300 cm^−1^ could be attributed to OH stretching (Hatamian et al. 2020; Kataria et al. 2022). It is possible to attribute the peaks detected around 3012 cm^−1^ to C=C–H stretching (García‐Salcedo et al. 2018). C–H stretching may be the reason for the peaks observed around 2950–2850 cm^−1^. C–H stretching of the chia samples was previously reported at 2933, 2922, 2872 cm^−1^ by Darwish et al. (2018), at 2922, 2852 cm^−1^ by Morales‐Olán et al. (2021) and at 3000–2800 cm^−1^ by Hatamian et al. (2020) and Noshad et al. (2020). The peak observed around 1745–1740 cm^−1^ for the chia samples could be attributed to C=O stretching (Darwish et al. 2018; García‐Salcedo et al. 2018; Hatamian et al. 2020; Noshad et al. 2020). The peak observed around 1600 cm^−1^ could be assigned to C=O stretching and Amide‐I group (secondary structure) (Carrión‐Prieto et al. 2017; Santos et al. 2023). The stretching of the carboxyl group (–COO–) of uronic acids may be the reason for the peaks observed around 1500 and 1460–1458 cm^−1^ (Timilsena et al. 2016; Noshad et al. 2020). The peak detected at 1245 cm^−1^ was attributed to the presence of Amide III (Hatamian et al. 2020; Noshad et al. 2020). The peak observed at 1038 cm^−1^ could be attributed to pyranose ring (García‐Salcedo et al. 2018).

The changes in the area of the peaks around 3300 cm^−1^ (OH stretching), 2950–2850 cm^−1^ (C–H stretching), and 1600 cm^−1^ (C=O stretching) might be due to the changes observed in the phenolic compounds of the chia after infrared treatment (Woranuch and Yoksan 2013; Morales‐Olán et al. 2021; Kataria et al. 2022). Protein structural alterations due to thermal denaturation might also be the reason for the changes observed in the peak around 1600 cm^−1^ (Ying 2015; Vanga et al. 2016).

### 3.4 Total Phenolic Content

Total phenolic content of the control and infrared treated chia samples was between 2.059 and 2.376 mg GAE/g dry sample (Table 3). Significantly (*p* < 0.05) higher total phenolic contents were observed for infrared treated samples (except 700 W–25 min) as compared to control. Total phenolic content increased significantly as the infrared power increased for each treatment time. For each infrared power, total phenolic content increased significantly as the infrared treatment time increased from 25 to 50 min. Among all samples, the highest total phenolic content (2.376 mg GAE/g dry sample) was obtained for the chia sample infrared treated at 1100 W for 50 min. Similar increases by using infrared treatment were also reported by Yalcin and Basman (2016) for total phenolic contents of soybean samples.

**TABLE 3:** Total phenolic content, total flavonoid content and antioxidant capacity of the infrared treated chia samples.

Sample	Total phenolic content (mg GAE/g, db)	Total flavonoid content (mg QE/g, db)	Antioxidant capacity	
			Radical scavenging activity (%)	Total antioxidant capacity (TAC, mmol TE/kg sample)
Control	2.059 ± 0.009^e^	1.157 ± 0.001^bc^	75.11 ± 0.15^f^	83.89 ± 0.45^g^
700 W–25 min	2.065 ± 0.013^e^	1.174 ± 0.048^bc^	76.79 ± 0.31^e^	118.24 ± 0.22^f^
700 W–50 min	2.097 ± 0.013^d^	1.285 ± 0.055^a^	77.66 ± 0.18^d^	124.57 ± 0.22^e^
900 W–25 min	2.205 ± 0.021^c^	1.212 ± 0.033^b^	78.63 ± 0.15^c^	137.39 ± 0.45^d^
900 W–50 min	2.261 ± 0.014^b^	1.190 ± 0.007^b^	80.00 ± 0.15^b^	178.39 ± 0.22^c^
1100 W–25 min	2.262 ± 0.007^b^	1.147 ± 0.016^bc^	80.15 ± 0.15^b^	193.27 ± 0.22^b^
1100 W–50 min	2.376 ± 0.023^a^	1.121 ± 0.034^c^	80.71 ± 0.18^a^	211.63 ± 0.67^a^

*Note:* Values followed by the same letter in the same column are not significantly different (*α* = 0.05).

Higher total phenolic contents were obtained for control chia and infrared treated chia samples, as compared to the total phenolic contents reported for chia in the literature. The total phenolic content of chia seed was reported as 0.92 mg GAE/g (cv. Jalisco) and 0.88 mg GAE/g (cv. Sinaloa) (Reyes‐Caudillo et al. 2008), 97.7 mg GAE/100 g (db) (Beltrán‐Orozco et al. 2020), 1.6 mg GAE/g (Hatamian et al. 2020), 1.6398 mg GAE/g (Martínez‐Cruz and Paredes‐López 2014), 1.65 mg GAE/g dry sample (Khursheed et al. 2023), 2.14–2.21 mg GAE/g (white chia seeds), and 2.18–2.19 mg GAE/g (dark chia seeds) (Alvites‐Misajel et al. 2019).

Similar to our results, it was reported that the total phenolic contents of chia seeds increased from 3.07 mg GAE/g to 3.43 mg GAE/g by roasting at 120°C for 20 min in a tray oven (Ghafoor et al. 2018), from 349.44 mg GAE/100 g to 535 mg GAE/100 g by roasting at 120°C for 6 min in a teflon pan (Ghafoor et al. 2022), from 358 μg GAE/g to 538 μg GAE/g by roasting at 160°C–200°C for 5–15 min (Song et al. 2019), and from 186.59 mg/100 g to 429.29 mg/100 g by roasting in a teflon pan at 120°C for 5, 10, 15, and 20 min (Mohamed Ahmed et al. 2024). The reason for the increase in the total phenolic content of chia seeds may be the products formed as a result of nonenzymatic browning due to prolonged exposure to high temperatures. The increase in total phenolic content can also be attributed to the disruption of the cells containing phenolic compounds and the improved extractability of bound phenolics through thermal treatment (Haripriya 2020; Mohamed Ahmed et al. 2024).

Hatamian et al. (2020) reported variations in the total phenolic content of chia seeds roasted at 160°C and 180°C. Roasting at 160°C for 15–35 min increased the content from 1.6 mg GAE/g (control) to a maximum of 2.7 mg GAE/g. However, roasting at 180°C initially led to a decrease, but the highest value (3.2 mg GAE/g) was observed at 35 min.

In contrast to the results of our study, a decrease in total phenolic content of chia seeds was reported. A decrease from 0.98 to 0.91 (90°C), 0.88 (120°C), 0.76 (150°C), and 0.51 mg GAE/100 g (180°C) was reported for roasting of chia seeds for 30 min (Ghafoor et al. 2020). Al‐Juhaimi et al. (2024) also reported a decrease from 2.55 to 2.34 mg GAE/g by roasting of chia seeds at 90°C for 1 h and to 2.14 mg GAE/g by roasting at 120°C for 30 min.

### 3.5 Total Flavonoid Content

The total flavonoid content of the control chia was 1.157 mg quercetin (QE)/g dry sample (Table 3). Infrared treatment at 700 and 900 W resulted in an increase in total flavonoid content of the chia samples. Among all samples, the significantly highest flavonoid content (1.285 mg quercetin [QE]/g dry sample) was obtained for the chia sample infrared treated at 700 W for 50 min. Improved extractability of flavonoids through the thermal breakdown of cellular components may lead to an increase in total flavonoid content of the chia seeds. Infrared treatment at 1100 W (for 25 min or 50 min) caused a decrease in total flavonoid content.

The total flavonoid content of the chia seeds used in this study was within the range of values reported in the literature for chia. The total flavonoid content was reported as 35.8 mg QE/100 g (db) (Beltrán‐Orozco et al. 2020), 0.35 mg QE/g dry sample (Khursheed et al. 2023), 1.50–1.56 mg CE/g (white chia seeds), 1.54–1.57 mg CE/g (dark chia seeds) (Alvites‐Misajel et al. 2019), and 2.21 mg CE/g (Ghafoor et al. 2018). The total flavonoid content of chia seeds extracted at different temperatures (40°C, 50°C, 60°C) was reported as 0.110–0.162 g/kg QE (Scapin et al. 2016). Similar to the results obtained for infrared treatment used in the present study, variations in the total flavonoid content of chia seeds have also been reported for roasting in the literature. The total flavonoid content of chia seeds increased from 286 to 421 μg QE/g by roasting at 160°C, 180°C, 200°C for 5, 10, 15 min (Song et al. 2019) and from 2.21 to 2.51 mg CE/g by roasting at 120°C–20 min in an oven (Ghafoor et al. 2018). Total flavonoid content decreased from 17.62 to 9.86 mg CE/100 g by roasting of chia seeds at 90°C, 120°C, 150°C, and 180°C (Ghafoor et al. 2020), and from 1217.62 to 1020.48 mg QE/100 g by roasting at 120°C for 6 min in a teflon pan (Ghafoor et al. 2022). Al‐Juhaimi et al. (2024) also reported a decrease from 14.61 to 13.71 mg CE/g (90°C for 1 h) and to 12.91 mg CE/g (120°C for 30 min) for roasted chia seeds. In a study by Mohamed Ahmed et al. (2024), an increase from 900.48 (control) to 903.33 mg/100 g (5 min) and a decrease from 900.48 (control) to 810.00 mg/100 g (10 min), 850.95 mg/100 g (15 min), and 886.19 mg/100 g (20 min) was reported for roasting chia seeds at 120°C. It has been stated that the decrease in total flavonoid content over time due to thermal processing may be attributed to the degradation of flavonoid compounds in chia seeds (Mohamed Ahmed et al. 2024).

### 3.6 Antioxidant Capacity

#### 3.6.1 Radical Scavenging Activity (DPPH)

The radical scavenging activities of the control and infrared treated chia samples were between 75.11% and 80.71% (Table 3). Infrared treatment caused a significant increase in radical scavenging activities of the chia samples. The significantly highest (80.71%) radical scavenging activity was obtained for the chia sample infrared treated at 1100 W for 50 min. It was observed that radical scavenging activities increased significantly either as the infrared power increased for each treatment time or as the treatment time increased for each infrared power.

High antioxidant activity of chia was due to the presence of phenolic substances, such as phenolic acids, isoflavones, and anthocyanins (Martínez‐Cruz and Paredes‐López 2014). In literature, the radical scavenging activities of the chia seeds were reported as 30% (Hatamian et al. 2020), 65.44% (Al‐Juhaimi et al. 2024), 68.83% (Martínez‐Cruz and Paredes‐López 2014), and 88.27% (Ghafoor et al. 2020).

Hatamian et al. (2020) and Al‐Juhaimi et al. (2024) reported an increase in radical scavenging activity by roasting of chia seeds. Hatamian et al. (2020) reported an increase from 30% (control) to 61% (180°C–25 min), whereas Al‐Juhaimi et al. (2024) found an increase from 65.44% (control) to 80.13% (90°C–1 h) and 75.84% (120°C–30 min). However, Ghafoor et al. (2020) reported a decrease from 88.27% (control) to 85.76% (90°C), 79.57% (120°C), 48.44% (150°C), and 31.23% (180°C). Mohamed Ahmed et al. (2024) also reported a decrease in antioxidant activity of chia seeds by roasting in a teflon pan at 120°C for 10–20 min.

#### 3.6.2 Total Antioxidant Capacity (QUENCHER‐CUPRAC)

Total antioxidant capacity (TAC) of the infrared treated chia samples (118.24–211.63 mmol TE/kg sample) was significantly higher than that of the control (83.89 mmol TE/kg sample) (Table 3). Among all chia samples, the significantly highest (211.63 mmol TE/kg sample) TAC value was obtained for the chia sample infrared treated at 1100 W for 50 min. It was observed that TAC values increased significantly either as the infrared power increased for each treatment time or as the treatment time increased for each infrared power.

The TAC values found for chia were higher as compared to the TAC values reported in literature for cereals; wheat germ (40.22 ± 1.86 mmol TE/kg sample), barley (26.35 ± 0.61 mmol TE/kg sample), rye (16.21 ± 0.25 mmol TE/kg sample), sorghum (13.55 ± 0.55 mmol TE/kg sample), wheat (13.44 ± 0.46 mmol TE/kg sample), and oat (10.46 ± 0.23 mmol TE/kg sample) (Tufan 2012).

### 3.7 Phenolic Profile

Some phenolic and flavonoid compounds determined by HPLC analysis of the methanolic extracts of chia are given in Table 4. Infrared treatment caused a significant decrease in caffeic acid and p‐coumaric acid, whereas a significant increase was observed in chlorogenic acid, rutin, and ferulic acid. Quercetin content increased only at 700 W–50 min and 900 W–25 min. Improved extractability of phenolics through thermal breakdown of cellular components by the aid of infrared may lead to an increase in phenolics (chlorogenic acid, ferulic acid, rutin, quercetin) in chia.

**TABLE 4:** Phenolic profile of the infrared treated chia samples (mg/100 g, db).

Sample	Caffeic acid	p‐coumaric acid	Chlorogenic acid	Ferulic acid	Rutin	Quercetin
Control	1.01 ± 0.01^a^	5.27 ± 0.24^a^	2.40 ± 0.02^g^	ND	ND	20.58 ± 0.29^bc^
700 W–25 min	0.84 ± 0.02^b^	4.62 ± 0.07^b^	3.76 ± 0.07^f^	0.82 ± 0.02^e^	ND	20.36 ± 0.41^c^
700 W**–**50 min	0.85 ± 0.01^b^	4.49 ± 0.02^b^	4.72 ± 0.07^e^	1.02 ± 0.08^d^	3.91 ± 0.09^e^	21.10 ± 0.34^a^
900 W**–**25 min	0.68 ± 0.04^c^	2.66 ± 0.02^c^	9.36 ± 0.10^d^	1.81 ± 0.12^b^	6.06 ± 0.07^d^	20.79 ± 0.27^ab^
900 W**–**50 min	0.66 ± 0.02^c^	1.86 ± 0.02^d^	9.84 ± 0.14^c^	1.88 ± 0.17^b^	10.32 ± 0.36^c^	19.25 ± 0.09^d^
1100 W**–**25 min	0.51 ± 0.02^d^	0.62 ± 0.06^e^	11.45 ± 0.11^b^	2.15 ± 0.14^a^	15.27 ± 0.18^b^	18.46 ± 0.17^e^
1100 W**–**50 min	ND	ND	11.78 ± 0.03^a^	1.44 ± 0.07^c^	26.48 ± 0.91^a^	12.53 ± 0.03^f^

*Note:* Values followed by the same letter in the same column are not significantly different (*α* = 0.05). Values are the means of four replicates.

Abbreviations: db, dry basis; ND, not detected.

Caffeic acid content of control chia (1.01 mg/100 g [db]) was significantly higher compared to those of infrared treated samples. It was observed that the amount of caffeic acid decreased significantly as the infrared power increased for each treatment time, except for 1100 W–50 min chia. The caffeic acid was not detected for the chia infrared treated at 1100 W for 50 min. No significant change was observed in caffeic acid when treatment time increased from 25 to 50 min at 700 and 900 W. Caffeic acid content of the chia seeds used in this study was within the range of values reported in the literature for chia. Caffeic acid content of chia was reported as 30.89 μg/g (Coelho and Salas‐Mellado 2014), 0.0030 mg/g seed (cv. Jalisco) and 0.00680 mg/g seed (cv. Sinaloa) (Reyes‐Caudillo et al. 2008), 0.45 mg/100 g (db) (Pająk et al. 2019), 1.52 mg/100 g (Al‐Juhaimi et al. 2024), 1.75 mg/100 g (Ghafoor et al. 2022), 31.14 mg/kg (Ghafoor et al. 2018), and 0.280 mg/g (db) (Abdel‐Aty et al. 2021).

Infrared treatment caused a significant decrease in p‐coumaric acid content of chia. For each infrared treatment time (25 or 50 min), as the infrared power increased, the amount of p‐coumaric acid decreased significantly, except for 1100 W–50 min chia. p‐coumaric acid was not detected in the chia sample infrared treated at 1100 W for 50 min. Previous studies reported lower p‐coumaric acid content (2.93 mg/100 g, Ghafoor et al. (2022) and 4.17 mg/kg Ghafoor et al. (2018)) for chia.

Chlorogenic acid content of control chia was found to be 2.40 mg/100 g (db). As the infrared power and time increased, chlorogenic acid significantly increased. Among all samples, the significantly highest (11.78 mg/100 g [db]) chlorogenic acid content was obtained for the 1100 W–50 min chia sample. In the literature, chlorogenic acid content of the chia was reported as 4.68μg/g (Coelho and Salas‐Mellado 2014), 0.028 mg/g (db) (Abdel‐Aty et al. 2021), 0.102 mg/g seed (cv. Jalisco) and 0.0459 mg/g seed (cv. Sinaloa) (Reyes‐Caudillo et al. 2008).

Infrared treatment led to the detection of ferulic acid, which was not found in the control chia sample. It was observed that the ferulic acid content increased significantly as the infrared power increased for each treatment time, except for the 1100 W–50 min treatment. The highest (2.15 mg/100 g, db) ferulic acid content was observed in the 1100 W–25 min chia. Ferulic acid content of chia was reported as 2.14 mg/kg (Ghafoor et al. 2018), 0.16 mg/100 g (db) (Pająk et al. 2019), 0.54 mg/100 g (Ghafoor et al. 2022), and 0.112 mg/g (db) (Abdel‐Aty et al. 2021).

Rutin was not detected in the control and 700 W–25 min chia. The extraction of rutin was initially achievable with the infrared treatment at 700 W for 50 min. The highest (26.48 mg/100 g [db]) rutin content was observed in the chia infrared treated at 1100 W for 50 min. The amount of rutin increased significantly at 900 W or 1100 W as the infrared treatment time increased from 25 to 50 min. Ghafoor et al. (2022) reported rutin content of chia as 3.98 mg/100 g.

Quercetin is the mostly known flavonoid in chia. Quercetin content of control chia was found to be 20.58 mg/100 g (db). Significantly highest (21.10 mg/100 g, db) quercetin content was found in the chia infrared treated at 700 W for 50 min. Insignificant change was observed for the 900 W–25 min sample (20.79 mg/100 g, db), as compared to the 700 W–50 min sample. Higher quercetin content was obtained for chia used in the study as compared to the values (0.17 μg/g, Coelho and Salas‐Mellado (2014); 10.85 mg/100 g, Al‐Juhaimi et al. (2024); 5.01 mg/100 g Ghafoor et al. (2022); and 0.080 mg/g (db) Abdel‐Aty et al. (2021)).

Similar to the results obtained in the present study, a decrease in caffeic acid (from 1.75 to 0.50 mg/100 g) and p‐coumaric acid (from 2.93 to 1.28 mg/100 g), as well as an increase in rutin (from 3.98 to 24.92 mg/100 g) content was also reported by Ghafoor et al. (2022) for chia samples roasted at 120°C for 6 min in a teflon pan. On the other hand, Ghafoor et al. (2018) reported an increase in caffeic acid (from 31.14 to 35.46 mg/kg) and p‐coumaric acid (from 4.17 to 5.48 mg/kg) of chia samples roasted at 120°C for 20 min in an oven. Similar to the increase in ferulic acid content observed with infrared treatment of chia seeds, Ghafoor et al. (2018) also reported an increase after the roasting of chia seeds at 120°C for 20 min. However, in a study by Ghafoor et al. (2022) it was found that roasting at 120°C for 6 min in a teflon pan had no effect on ferulic acid content. Mohamed Ahmed et al. (2024) reported variations in the caffeic acid and rutin content of chia seeds roasted at 120°C. Roasting at 120°C for 5–10 min caused a decrease in caffeic acid and rutin content. However, roasting at 120°C for 15–20 min led to an increase. A decrease followed by an increase (only at 20 min) was reported for p‐coumaric acid, ferulic acid, and quercetin. It was stated that the decrease followed by an increase could be due to the breakdown of free phenolics and the subsequent release of bound phenolics over time (Mohamed Ahmed et al. 2024). Al‐Juhaimi et al. (2024) reported that roasting of chia (90°C–1 h or 120°C–30 min) resulted in an increase in caffeic acid and rutin content but a decrease in quercetin content. In the study by Kaur et al. (2025), infrared pretreatment (140°C, 160°C, and 180°C for 5 and 10 min) of linseeds resulted in an increase in rutin content from 0.64 (control) to 4.29 mg/100 g and a decrease in quercetin content from 0.89 (control) to 0.58 mg/100 g.

### 3.8 Functional Properties

Chia can be used as a natural ingredient in food formulations due to water and oil holding capacity, emulsion activity, foaming stability, and gel formation (Guedes de Melo et al. 2022). The water holding capacity of chia samples ranged from 1.82 to 8.44 g water/g dry sample, with the highest value (8.44 g/g) in the control chia (Table 5). A significant (*p* < 0.05) decrease in water holding capacity was observed as the infrared power and time increased. Water holding capacity decreased significantly either as the infrared power increased for each treatment time or as the treatment time increased from 25 to 50 min for each infrared power.

**TABLE 5:** Functional properties of the infrared treated chia samples.

Sample	Water holding capacity (g water/g dry sample)	Oil holding capacity (g oil/g dry sample)	Emulsion activity (%)	Emulsion stability (%)
Control	8.44 ± 0.05^a^	1.94 ± 0.03^a^	20.46 ± 0.22^d^	9.91 ± 0.18^c^
700 W–25 min	7.61 ± 0.09^b^	1.89 ± 0.03^b^	20.73 ± 0.02^c^	11.27 ± 0.09^b^
700 W–50 min	5.37 ± 0.05^c^	1.85 ± 0.02^c^	22.68 ± 0.19^a^	12.00 ± 0.27^a^
900 W–25 min	3.37 ± 0.04^d^	1.82 ± 0.01^cd^	22.27 ± 0.09^b^	8.12 ± 0.18^d^
900 W–50 min	2.30 ± 0.10^e^	1.80 ± 0.02^de^	6.98 ± 0.10^e^	2.93 ± 0.20^e^
1100 W–25 min	2.12 ± 0.01^f^	1.78 ± 0.02^e^	2.62 ± 0.10^f^	1.94 ± 0.10^f^
1100 W–50 min	1.82 ± 0.08^g^	1.72 ± 0.01^f^	1.90 ± 0.09^g^	1.29 ± 0.10^g^

*Note:* Values followed by the same letter in the same column are not significantly different (*α* = 0.05). Values are the means of three replicates.

The oil holding capacity of the chia samples was between 1.72 and 1.94 g oil/g dry sample. The control chia had a significantly higher oil holding capacity as compared to that of infrared treated samples. Infrared treatment caused a slight but significant decrease in the oil holding capacity of the chia samples.

Higher water holding capacity was obtained for control chia sample in the present study as compared to the values reported in literature. Water holding capacity of chia was reported as 5 g water/g sample (for whole chia seed), 5.5 g water/g sample (for chia seeds powder) (Darwish et al. 2018), 6 g/100 g (Hatamian et al. 2020). The oil holding capacity of the chia used in this study was within the range of the values reported in the literature for chia. Oil holding capacity of chia seed was reported as 3.9 g/100 g (Hatamian et al. 2020), 2.23 mL/g (García‐Salcedo et al. 2018), 1.8 mL/g (Haripriya 2020), 3.5 g oil/g sample (for whole chia seed), 2.5 g oil/g sample (for chia seeds powder) (Darwish et al. 2018).

Similar to our results, a decrease in water absorption and oil absorption capacity was also reported for chia roasted in a griddle for 5–7 min (Haripriya 2020). A decrease in water absorption capacity from 5.2 to 2.7 mL/g and in oil absorption capacity from 1.8 to 1.2 mL/g was reported by Haripriya (2020). Song et al. (2019) also reported a decrease in water holding capacity of chia seeds roasted at 160°C, 180°C, and 200°C for 5, 10, and 15 min (except 180°C–5 min). In contrast to the decrease in water and oil absorption capacity by infrared treatment used in the present study, an increase in water absorption capacity (from 6 to 6.4 g/100 g) and in oil absorption capacity (from 3.9 to 5.3 g/100 g) was reported by Hatamian et al. (2020) for chia seeds roasted at 180°C.

Internal factors, such as the composition of amino acids, the shape of the protein, and the degree of polarity or hydrophobicity on the surface affect the ability of food protein to bind water and oil. The water absorption capacity is strongly associated with the quantity of amino acids and the presence of available protein functional groups within the chia (Haripriya 2020). The changes in FTIR spectra related to the protein, especially for the peak at 1636 cm^−1^, might cause variations in the water holding capacities of the infrared treated samples.

The emulsion activity of chia samples ranged from 1.90% to 22.68%, with the significantly highest value (22.68%) observed in the 700 W–50 min sample. Compared to the control, the 700 W–25 min, 700 W–50 min, and 900 W–25 min samples showed significantly higher emulsion activity, whereas the 900 W–50 min, 1100 W–25 min, and 1100 W–50 min samples showed significantly lower values.

The emulsion stability of the chia samples was between 1.29% and 12.00%. Among all chia samples, similarly to emulsion activity value, the significantly highest emulsion stability (12.00%) was obtained for the 700 W–50 min chia sample. As the treatment time increased from 25 to 50 min, infrared treatment at 700 W caused a significant increase in emulsion stability, whereas infrared treatment at 900 and 1100 W caused a significant decrease.

Guldiken et al. (2022) also reported higher emulsion capacity for infrared treatment of tempered navy bean and chickpea flour. The increase was attributed to increased balanced surface ability of hydrophobic and hydrophilic sites of proteins.

## 4 Conclusions

Infrared treatment at all powers and times caused a significant increase in total phenolic content and antioxidant capacity (DPPH and TAC) of chia samples. Significantly highest values were obtained for the chia sample infrared treated at 1100 W for 50 min. Similar increases were also reported in literature for roasted chia seeds. Infrared treatment at 700 and 900 W caused an increase in total flavonoid content. Infrared treatment caused a significant increase in chlorogenic acid. Ferulic acid and rutin were not detected in the control chia sample. Infrared treatment enabled the extraction of rutin and ferulic acid. A significant increase was observed in quercetin content of 700 W–50 min and 900 W–25 min chia samples. Improved extractability through the thermal breakdown of cellular components by the aid of infrared treatment may result in an increase in phenolics and flavonoids. This increase may contribute to antioxidant capacity. Infrared treatment at 700 and 900 W caused an increase in total dietary fiber content. Emulsion activity and emulsion stability significantly increased with infrared treatment up to 900 W–25 min and 700 W–50 min, respectively. Infrared treatment caused a significant decrease in water and oil holding capacity. Compatible results have been reported in literature for the roasting of chia. FTIR spectrums for infrared treated chia samples include all the specific peaks reported in literature for chia, but slight changes were observed in the intensity of some of the peaks. The changes in the peaks related to protein might be one of the reasons for the changes in the functional properties of the infrared treated chia samples.

Chia has high nutritional value and a positive impact on health due to its higher phenolics and flavonoids, essential oils, protein, and total dietary fiber. Infrared treatment applied to chia resulted in an increase in its health‐beneficial components. Infrared treatment shows great potential for the utilization of chia in value‐added food products for increasing the consumability of chia seeds with improved nutritional quality, taste, and aroma.

## Author Contributions

**Meltem Laçin:** conceptualization (equal), methodology (equal), investigation (equal), writing – original draft (equal), formal analysis (lead), visualization (lead). **Arzu Başman:** conceptualization (equal), methodology (equal), investigation (equal), writing – original draft (equal), writing – review and editing (lead), supervision (lead), project administration (lead).

## Conflicts of Interest

The authors declare no conflicts of interest.

## Supporting information


Data S1.

Data S2.

## Data Availability

Data will be made available on request.
