# Supplementary material for: Effects of Infrared Treatment on Some Constituents and Functional Properties of Chia Seed
Source: Food Sci Nutr. 2025 Jun 3;13(6):e70308. doi: 10.1002/fsn3.70308 (PMC12130633; doi:10.1002/fsn3.70308)

**Effects of infrared treatment on some constituents and**

**functional properties of chia seed**Meltem Laçin, Arzu Başman*


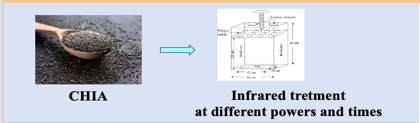


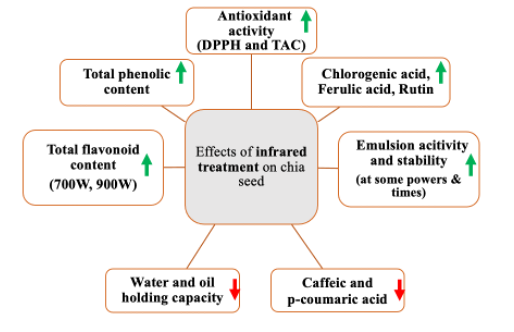

Supplement: Supplementary file 1 — Data S1. [file FSN3-13-e70308-s001.docx]
