# Supplementary material for: Effects of Infrared Treatment on Some Constituents and Functional Properties of Chia Seed
Source: Food Sci Nutr. 2025 Jun 3;13(6):e70308. doi: 10.1002/fsn3.70308 (PMC12130633; doi:10.1002/fsn3.70308)
Supplement: Supplementary file 2 — Data S2. [file FSN3-13-e70308-s002.docx]

Infrared treatment enhanced the antioxidant capacity, total flavonoid and total phenolic content of the chia and enabled the extractability of rutin and ferulic acid.

Infrared treatment adversely affected water and oil holding capacities while improved emulsion activity (at 700W and 900W-25min) and stability (at 700W).

*Infrared treatment which improves the amount of health beneficial constituents of chia is promising for utilization of chia as raw material in value-added food products.
